# Nonvolatile electrical switching of optical and valleytronic properties of interlayer excitons

**DOI:** 10.1038/s41377-022-00718-7

**Published:** 2022-01-24

**Authors:** Tong Ye, Yongzhuo Li, Junze Li, Hongzhi Shen, Junwen Ren, Cun-Zheng Ning, Dehui Li

**Affiliations:** 1 School of Optical and Electronic Information, Huazhong University of Science and Technology, 430074 Wuhan, China; 2 Wuhan National Laboratory for Optoelectronics, Huazhong University of Science and Technology, 430074 Wuhan, China; 3 Department of Electronic Engineering, Tsinghua University, 100084 Beijing, China; 4 Frontier Science Center for Quantum Information, 100084 Beijing, China; 5 School of Electrical, Computer, and Energy Engineering, Arizona State University, Tempe, AZ 85287 USA

**Keywords:** Photonic devices, Nanophotonics and plasmonics

## Abstract

Long-lived interlayer excitons (IXs) in van der Waals heterostructures (HSs) stacked by monolayer transition metal dichalcogenides (TMDs) carry valley-polarized information and thus could find promising applications in valleytronic devices. Current manipulation approaches for valley polarization of IXs are mainly limited in electrical field/doping, magnetic field or twist-angle engineering. Here, we demonstrate an electrochemical-doping method, which is efficient, in-situ and nonvolatile. We find the emission characteristics of IXs in WS_2_/WSe_2_ HSs exhibit a large excitonic/valley-polarized hysteresis upon cyclic-voltage sweeping, which is ascribed to the chemical-doping of O_2_/H_2_O redox couple trapped between WSe_2_ and substrate. Taking advantage of the large hysteresis, a nonvolatile valley-addressable memory is successfully demonstrated. The valley-polarized information can be non-volatilely switched by electrical gating with retention time exceeding 60 min. These findings open up an avenue for nonvolatile valley-addressable memory and could stimulate more investigations on valleytronic devices.

## Introduction

Van der Waals heterostructures (HSs) stacked by transition metal dichalcogenides (TMDs) monolayers enable the generation of long-lived interlayer excitons (IXs) with a large binding energy of about 150 meV^1^ and a long diffusion distance over five micrometers^2^, further extending the already appealing properties of the constituent TMDs monolayers. Since IXs are composed of electrons and holes that are resided in neighboring layers, their physical properties strongly depend on the layer configurations and external fields or dopings^3,4^. Through electrical field or doping, we can modulate the emission intensity and wavelength of the IXs^5^, and even switch its polarization^6^. Recently, IXs in the HSs stacked by other layered materials such as 2D perovskites and InSe with TMDs monolayer have been demonstrated and can be utilized in mid-infrared photodetections^7,8^.

In particular, IXs in TMDs-based heterostructures carry valley-polarized information and thus would find promising applications in valleytronics taking advantage of their long lifetime^9^. Previous studies have demonstrated that IXs exhibit a large valley-polarization degree that can be tuned in a wide range by external electric field^10^, magnetic field^11^, and twist-angle engineering^12^. Although considerable progress has been made in valleytronics, nonvolatile device that is indispensable for valleytronics has not been achieved up to date. Here, we demonstrate an IX-based nonvolatile valley-addressable memory, which would prompt relevant investigations on valleytronics.

## Results

**Fig. 1:**
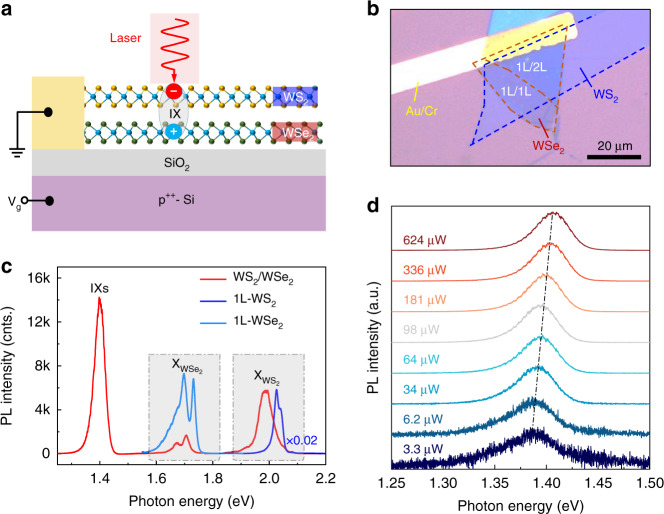
IXs in a WS_2_/WSe_2_ HS. **a**, **b** Schematic and optical microscope image of the device, respectively. 1L/1L and 1L/2L represents monolayer WS_2_/monolayer WSe_2_ and monolayer WS_2_/bi-layer WSe_2_ respectively. **c** PL spectra of the HS and monolayer WSe_2_ and WS_2_ at 78 K under 23 μW excitation at 532 nm. For a clear visualization, the PL spectrum of individual WS_2_ is multiplied by a factor 0.02. **d** PL spectra of the IXs as a function of excitation power under 633 nm laser excitation at 78 K.

In this work, the HS device is formed by a monolayer WS_2_ (top) and a monolayer WSe_2_ (bottom), both of which are contacted with an electrode (Fig. 1a). By applying voltage between the electrode and the heavily-doped Si substrate, we can control the doping level of the device when performing optical measurements. Figure 1b shows the optical microscope image of the device. The WS_2_ and WSe_2_ sheets are mechanically exfoliated from their respective bulk crystals and then transferred on a SiO_2_/p^++^-Si substrate through dry-transfer technique^13^. To minimize the generation of bubbles formed between the constituting monolayers, which would suppress the formation of interlayer excitons and thus weaken the switching behavior and chemical-doping effect discussed below, we adopted a tilt-transfer method (see “Materials and methods” section). The edges of the two sheets are also intentionally aligned to improve interlayer coupling^3^. The device also contains a monolayer WS_2_/bi-layer WSe_2_ HS region, which is labeled as 1L/2L to distinguish from the monolayer WS_2_/monolayer WSe_2_ (1L/1L) region. Since the two regions exhibit similar optical behaviors, for a simple discussion, the following text focuses on the measurements acquired in the 1L/1L region of the device, unless stated otherwise. The experimental data collected from the 1L/2L region is provided in supplementary materials (Fig. S1). The substrate was oxygen-plasma cleaned for 10 min before the dry-transfer procedure, so as to make a uniform hydrophilic surface^14^.

### IXs in the WS_2_/WSe_2_ HS

Figure 1c shows the PL spectra of the HS, from which we can observe severe PL quenching and redshift of the intralayer excitonic peaks, together with the appearance of a low-energy peak at 1.4 eV. The quenching and redshift of the intralayer excitonic peaks can be attributed to interlayer charge transfer^15,16^ and modified dielectric environment^17,18^, respectively. We assign the peak at 1.4 eV to the IX emission according to previous reports^19,20^. The excitation-power dependent PL spectra further verify its interlayer nature (Fig. 1d). The IX emission peak shows a monotonous blueshift with the increase of excitation power, which is due to many-body effect arising from the repulsive interaction between the dipole-aligned IXs^21,22^. Such monotonous behavior of IX also manifests that the laser-heating effect can be neglected during the measurements.

### Excitonic hysteresis of IXs

**Fig. 2:**
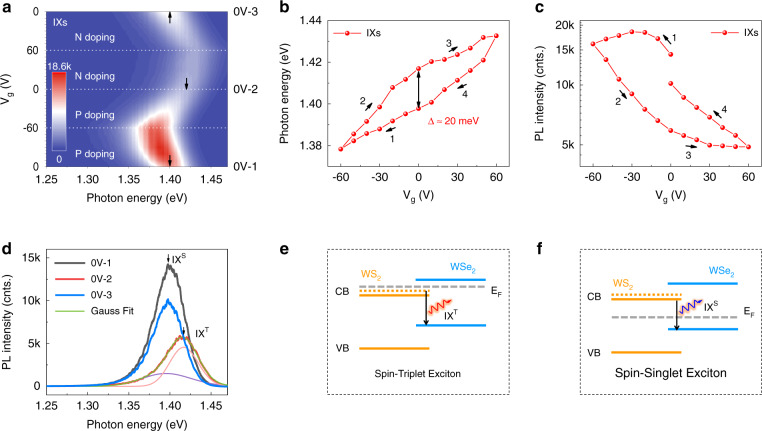
Electrical control of the IX emission. **a** Contour plot for the PL spectra of IXs upon cyclic *V*_g_. The white dashed lines serve as guides to the eye. Black arrows mark the peak positions of the IXs at 0 V with different scanning sequences. **b**, **c** Photon energy and PL intensity of the IX emission as a function of *V*_g_. **d** PL spectra of the IXs at 0 V with different scanning sequences. 0V-1, 0V-2, and 0V-3 represent three spectra marked in **a**. The 0V-2 spectrum is fitted by two Gaussian peaks. The purple and pink lines are attributed to the spin-singlet state (IX^S^) and spin-triplet state (IX^T^) of interlayer excitons, respectively. The sample was excited by a 532 nm laser with 23 μW power at 78 K. **e**, **f** Schematic of the spin-triplet and spin-singlet excitons. Chemical-doped electrons lift the Fermi level up and shift the IX^S^ to IX^T^. When those electrons are released, the IX^T^ return to IX^S^. The orange dashed line stands for the upper spin-splitting conduction band (CB) of WS_2_. Red and blue arrows represent the recombination paths of the IXs.

To explore gate-dependent features of the IX emission, we measured the PL spectra of the device under cyclic *V*_g_, which scans first from 0 to −60 V, then 0 V all the way to 60 V and finally back to 0 V (Fig. 2a). The IX emission peak shows a redshift and the emission intensity is enhanced with the decrease of *V*_g_, and vice versa. The redshift of the IX emission peak with *V*_g_ can be ascribed to the Stark effect^6^, which is verified by the opposite shift trend of the IX emission peak in devices with stacking order inversed (Fig. S2). Interestingly, the IX emission peak exhibits a strong hysteresis upon cyclic-voltage sweeping. As indicated by the black arrows in Fig. 2a, the peak energy of the IXs at middle 0 V (0V-2) cannot return to the same value of initial 0 V (0V-1), until a further upward scanning that is finally back to 0 V (0V-3). The gate-dependent photon energy and PL intensity can be seen more clearly in Fig. 2b, c. For a simple discussion, we only compare the states at 0V-2 and 0V-3. The photon energy of 0V-2 is blueshifted by about 20 meV with respect to that of 0V-3. Meanwhile, the PL intensity of 0V-2 is weaker than 0V-3 with a contrast ratio of about 1.7. It is worth to mention that the light intensity changes non-monotonously as *V*_g_ decreases from 0 to −60 V, indicating the occurrence of chemical doping^23–25^, which will be discussed in the following. The light intensity difference between 0V-1 and 0V-3 (Fig. 2c) might be due to different levels of chemical doping at the initial and final sweeping stages, because charge density can significantly influence the photoluminescence quantum yield of TMDs-based devices^26^.

As shown in Fig. 2d, the IX emission peak of 0V-2 can be decomposed to two Gaussian peaks (detailed fittings of the spectra are provided in Fig. S3). The energy difference of the two peaks is about 20 meV, which is consistent with the splitting energy of the conduction band of WS_2_ (see refs. ^27,28^), strongly suggesting the occurrence of spin-triplet excitons^29^. This peculiar phenomenon can be understood from the chemical-doping^23,24^ induced band-filling effect^6^, as depicted in Fig. 2e, f. When the device is chemically *n*-doped, the Fermi level will be lift up and IXs will shift to the spin-triplet state (IX^T^), which has an inefficient PL yield because of inversed spin. Contrarily, when the chemically-doped electrons are released, IXs will return to the spin-singlet state (IX^S^). Therefore, the IX emission peaks in 0V-1 and 0V-3 spectra are attributed to IX^S^ emission, and that in 0V-2 spectrum is mainly resulted from IX^T^. The IX^T^ and IX^S^ peaks can be well resolved in PL spectra acquired by picosecond laser excitation (Fig. S4a). In addition, the intensity ratio of IX^T^/IX^S^ increases with the increase of *V*_g_ (Fig. S4b), thus confirming the band-filling mechanism and IX^T^/IX^S^ origins. We have also measured the gate-dependent lifetime of the IXs (Fig. S4c–e). The lifetime of the IXs at 0V-2 is slightly shorter than at 0V-1 and 0V-3 rather than getting prolonged, further supporting the IX^T^/IX^S^ origins^11^.

### Mechanism of the excitonic hysteresis

Electrical hysteresis has been observed in devices based on two-dimensional materials, such as graphene and TMDs based field-effect transistors^30–33^. Generally, electrical hysteresis is attributed to the chemical-doping effect by doping species (O_2_ and H_2_O) that are bound at the device/substrate interface, and/or on the surface of the device^34–36^. In our case, we propose that the excitonic hysteresis mentioned above is originated from the same scenario.

**Fig. 3:**
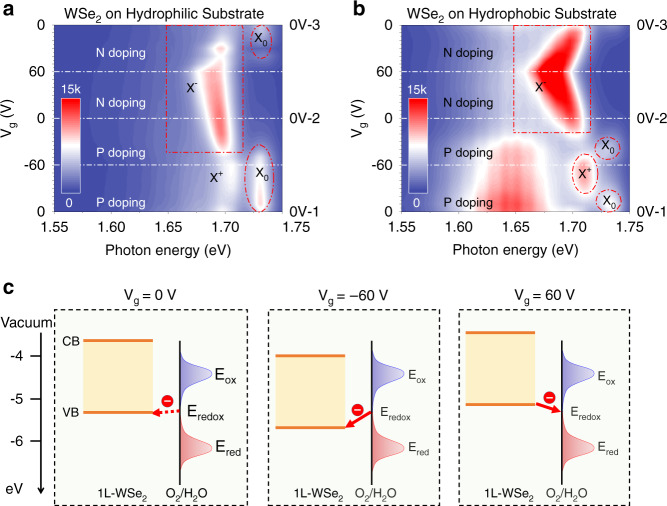
Mechanism of the excitonic hysteresis. **a** Contour plot for the PL spectra of monolayer WSe_2_ as a function of cyclic *V*_g_. The spectra were acquired in the individual WSe_2_ region of the HS on a hydrophilic substrate. **b** Contour plot for the PL spectra of monolayer WSe_2_ on a hydrophobic substrate, which is functionalized by hexamethyldisilazane (HDMS). The PL measurements were conducted at 78 K with 532 nm laser excitation (23 μW). **c** Illustration of chemical doping caused by O_2_/H_2_O molecules. The electronic density of states (DOS) reflect the electron energy distribution around the oxidation potential (*E*_ox_) and reduction potential (*E*_red_), respectively. *E*_redox_ is the energy where the DOS of reducing and oxidizing species are equal: *D*_ox_(*E*_redox_) = *D*_red_(*E*_redox_).

Since our measurements were performed in high vacuum (≈10^−7^ Torr), the influence of the molecules on the device surface can be safely neglected. Therefore, the excitonic hysteresis is more likely due to the O_2_/H_2_O molecules that are trapped at the interface between the HS and substrate. To clarify this, we examine the gate-dependent PL spectra of the individual WSe_2_ region (Fig. 3a), because WSe_2_ is in the bottom of the HS and directly contacts the SiO_2_/Si substrate.

The emission features of the intralayer excitons in WSe_2_ are closely correlated to that of IXs. As *V*_g_ decreases from 0 to −60 V (Fig. 3a), the emission of positive trions (X^+^) is gradually enhanced, while the peak of neutral excitons (X_0_) is suppressed, indicating an efficient hole doping (detailed data is provided in Fig. S5). Peculiarly, as *V*_g_ increases from −60 V back to 0 V, the evolution track is asymmetric to that from 0 to −60 V. The trion emission peak is firstly weakened, then enhanced and redshifted with the increase of *V*_g_. The asymmetric evolution strongly indicates the occurrence of negative trions (X^−^), and suggests that the WSe_2_ is chemically *n*-doped^36,37^ at 0V-2. When *V*_g_ increases from 0 to 60 V, the X^−^ peak is redshifted further, but with emission intensity weakened because of Coulomb screening from the free electrons^38^. When voltage scans backward from 60 to 0 V, the X^−^ peak shows a blueshift and the emission intensity becomes weaker while the X_0_ peak is gradually enhanced, indicating that the chemically-doped electrons have been released. All the above features are well consistent with the previously mentioned chemical-doping effect^23,24,36^.

To further validate such hypothesis, we conducted a control experiment with WSe_2_ monolayer on a hydrophobic substrate (Fig. 3b). The evolution tracks of X^+^ and *X*_0_ emission are roughly symmetrical along the black dashed line at about −60 V. The slight deviation might be due to trace O_2_/H_2_O molecules that are adsorbed on WSe_2_ before the transfer procedure. Besides, in sharp contrast to Fig. 3a, the track of X^−^ is quasi-symmetrical along the dashed line at 60 V, suggesting that the excitonic hysteresis is largely suppressed. Therefore, H_2_O molecules should play a critical role in our observations. The unknown peak centered at about 1.65 eV probably arises from dark states, charged dark states and phonon-assisted sideband emission from the dark excitons^39,40^, which require further investigations.

The surface of SiO_2_ is usually covered with a layer of silanol groups (≡Si–OH), especially after it is treated by piranha solution or plasma cleaner^23,36^. With these silanol groups, SiO_2_/Si substrates are easily bound by ambient O_2_ and H_2_O molecules^36^. As shown in Fig. 3c, the chemical potential of the redox couple (O_2_/H_2_O) is about −5.3 eV^23,41^, which is slightly higher than the valence band of WSe_2_ (about −5.46 eV)^42,43^. Therefore, electrons spontaneously transfer from O_2_/H_2_O to WSe_2_, making monolayer WSe_2_ initially *n*-doped (detailed information is provided in Fig. S5), and resulting in the asymmetry evolution of *X*_0_ in Fig. 3a.

When applying negative gate voltages, electrons are forced to transfer further from O_2_/H_2_O to WSe_2_. The chemical-doped electrons are trapped in WSe_2_ when *V*_g_ returns from −60 to 0 V, because the chemical-potential barrier between WSe_2_ and O_2_/H_2_O block electrons out (detailed supporting data can be seen in Fig. S6). Consequently, the Fermi level of the HS is lifted up, and IXs shift to the spin-triplet state (Fig. 2e) due to the band-filling mechanism^4,6^. With Fermi level raised up, photon-excited electrons are driven into the upper spin-flipping level due to Coulomb blocking effect. Those chemically-doped electrons balance out the gate modulation, resulting in the non-monotonic behavior of the IXs in 0~−60 V range (Fig. 2c) and the excitonic hysteresis. The chemical-doping effect also explains why X_0_ emission maintains its intensity from 0 to −60 V for WSe_2_ on the hydrophilic substrate (Fig. 3a) but greatly suppressed on the hydrophobic substrate (Fig. 3b).

When applying positive gate voltages, the chemical-doped electrons are driven back from WSe_2_ to the O_2_/H_2_O redox couple. Therefore, IXs return to the spin-singlet state (Fig. 2f) when *V*_g_ scans back to 0V-3. This control experiment further verifies the chemical-doping mechanism and well explains the origin of the excitonic hysteresis of IXs shown in Fig. 2. The hysteresis is largely suppressed in HSs stacked on hydrophobic substrates (Fig. S7). To demonstrate this, we have also fabricated WS_2_/WSe_2_/*h*BN HSs on hydrophilic substrates with WS_2_/WSe_2_ HS partially separated from the substrate by *h*BN with a thickness of around 50 nm. For these devices, the excitonic hysteresis is observed in the region where WS_2_/WSe_2_ HS directly contacts with the substrate, but absent in the *h*BN-insulated region (Fig. S8), further supporting the chemical-doping mechanism. Additionally, the area of the hysteresis curve is in proportion to the chemical-doping level, and could be quantitatively controlled by oxygen-plasma-cleaning time of the SiO_2_/Si substrate as demonstrated in WSe_2_-based memories^14^. The hysteretic behavior is re-confirmed by two-cycle-scanning measurements (Fig. S9), and well reproducible in multiple repeating measurements and also in different samples. Therefore, we rule out the influence of random contamination.

### Valley-polarized hysteresis of the IXs

**Fig. 4:**
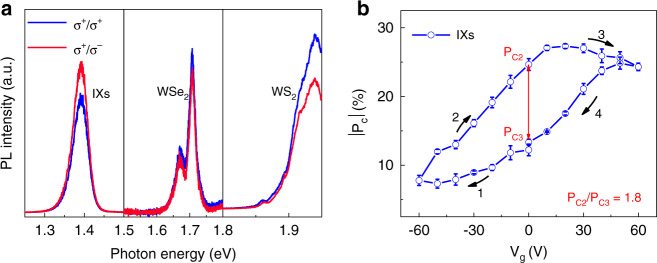
Electrically-tunable valley polarization of the IXs. **a** Helicity-resolved PL spectra of the HS under 633 nm excitation (180 μW) at 78 K. **b** Absolute circular polarization degree of the IXs as a function of *V*_g_. The helicity contrast is defined as ${P}_{{\mathrm{c}}2}/{P}_{{\mathrm{c}}3}$, where ${P}_{{\mathrm{c}}2}$ and ${P}_{{\mathrm{c}}3}$ is the absolute circular polarization degree at 0V-2 and 0V-3, respectively. The error bars in **b** represent the uncertainty of the data extraction

To study the chemical-doping effect on the valley-polarized features of the IXs, we measured the helicity-resolved PL spectra of the device (Fig. 4a). Interestingly, the IX peak exhibits a negative circular polarization in contrast to that of intralayer excitons in WSe_2_ and WS_2_, which can be ascribed to the interlayer quantum interference imposed by the atomic registry between the constituent layers^44^. To qualify the valley polarization, the degree of circular polarization (DOCP) has been introduced and defined as *P*_c_ = (*I*^+^ − *I*^−^)/(*I*^+^ − *I*^−^), where *I*^+^ (*I*^−^) denotes the intensity of co-polarized (cross-polarized) PL component. For the IXs peak, *P*_c_ = −12.3%, while for the intralayer excitonic peak of WS_2_ and WSe_2_, *P*_c_ = 15% and 7.1%, respectively. It is worth to mention that the helicity-resolved measurement was performed at 78 K with excitation power of about 180 μW. Therefore, the spatial modulation of moiré potential on optical selection rules for interlayer excitons can be neglected, since the moiré trapping effect can only be observed with ultra-low power excitation at ultra-low temperature^45^.

The DOCP of the IXs can also be electrically controlled by *V*_g_, as shown in Fig. 4b (the full data set is provided in Fig. S10). The absolute DOCP is greatly suppressed at −60 V (*p*-doping), but enhanced at 60 V (*n*-doping). This phenomenon has been reported by *Scuri* and coworkers, and is attributed to changes in valley-depolarization time caused by electron/hole doping^12^. Similarly, we believe our observations can be also ascribed to the charge doping from external applied bias and chemical doping (Fig. S4). Interestingly, the DOCP and lifetime (Fig. S4e) of the IXs also exhibit a strong hysteresis, probably due to the carrier trapping and detrapping induced by the above-mentioned chemical-doping, which leads to different doping concentrations and further different valley-depolarization time and DOCP under the same gate voltage. To sum up, the chemical-doping effect leads to the formation of spin-triplet excitons, and gives rise to the hysteresis of excitonic emission, valley-polarization degree and lifetime of IXs, which could find potential applications in nonvolatile valley-dependent information processing.

### IX-based valley-addressable memory

**Fig. 5:**
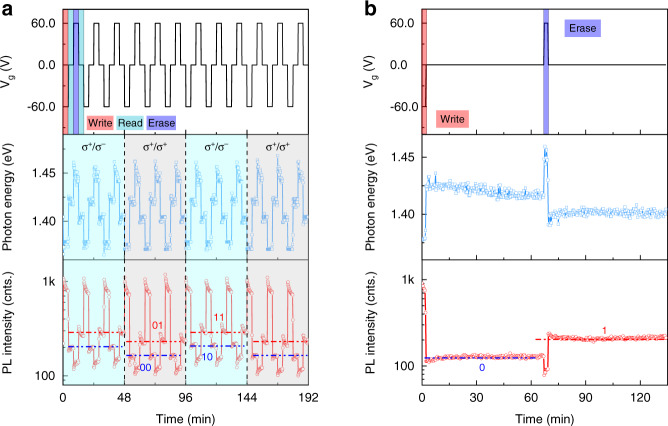
Electrically controlled memory operations in the HS. **a** Time-dependent IX emission characteristics upon cyclic *V*_g_ among −60, 0, 60, and 0 V. Each voltage lasts for about 4 min. The detecting polarization shifts every three cycles of *V*_g_. **b** Retention time of the “1” and “0” excitonic states. The writing and erasing voltages last for about 3 min, and the reading voltage lasts for about 64 min. The peak energies and intensities are extracted from real-time spectra, each of which was measured within 10 s. The sample was excited by a 633 nm laser with a power of 180 μW at 78 K.

To demonstrate the valley-encoding ability of the device, we measured time-dependent PL spectra under circular excitation (σ^+^), as shown in Fig. 5a. As gate voltage cyclically changes among −60, 0, 60, and 0 V, the photon energy of the IX emission periodically shifts among 1.38, 1.42, 1.45, and 1.40 eV, which are analogous to the performance of conventional electronic devices under “write”, “read”, and “erase” operations. In addition, the emission intensity also periodically changes in response to those memory operations. Specifically, the intensity level of IX^S^ (IX^T^) located at 1.40 (1.42) eV can be regarded as digital information 1 (0), which can persist for a long time with no power consumption, suggesting potential applications in nonvolatile storage. Intriguingly, as the detection helicity switches between σ^−^ and σ^+^, the PL intensity of the 0 and 1 states exhibit helicity-resolved features. There are four intensity levels emerging, which can be defined as “00”, “01”, “10”, and “11”, indicating valley-encoding abilities of the device. Based on this feature, we can selectively encode/address the valley-polarized information by helicity excitation/detection.

To evaluate the retention time of the encoded information, we then prolong the reading-operation time, as shown in Fig. 5b. Surprisingly, the 1 and 0 excitonic states can persist for at least 60 min, holding great promise for nonvolatile valley-addressable memory. As a matter of fact, the retention time should be much longer than 60 min, as can be seen in a logarithmic-timescale plot (Fig. S11a). This long retention time can be attributed to the synergetic blocking effect of the type-II band alignment of the HS and the chemical-potential barrier between WSe_2_ and O_2_/H_2_O (Fig. S6). We also note that the 0 (1) state varies dynamically before reaching a steady state. This is probably due to the charging/discharging process of the device, as confirmed by the features of time-dependent gate current (Fig. S11b). Importantly, the performance of the device is so robust that it can immune laser-heating effect, since the laser was kept focusing on the sample during the measurements. In addition, the information encoding ability of the device can persist up to about 250 K, which is promising for high temperature valleytronic applications (Fig. S12).

Since the nonvolatile valley-addressable memory has never been reported, it is hard to make an objective comparison. Nevertheless, the device is similar to photonic memory, thus we list the parameters of our device and other nonvolatile photonic memories in Table 1, which shows that our device is outperforming in comparison with peer memory devices. The PL ON/OFF ratio of the 1/0 states could be as large as 3.6 (Fig. S13), which is larger than peer photonic memories^46–49^. The power consumption of the device is estimated to be about 74/56 nW for set/reset operation (Fig. S11b), which is extremely low in comparison with other phase-change photonic memories^46–49^. The switching time of our devices could be very short but limited by our testing system, since the hysteresis effect could be established in several microseconds according to previous reports^50^.

**Table 1:** Parameters of our device and peer works

Memory type	ON/OFF ratio	Operation time (ns)	Power (Set/Reset) (mW)	Ref.
All-photonic	1.21	1	53.3 (O.P.)	^46^
All-photonic	1.8	5	10/30 (O.P.)	^49^
Ele-photonic	3.16	80100	10/110 (E.P.)	^47^
Ele-photonic	1.04	510 (E.P.) 408 (O.P.)	0.03/1.2 (E.P.) 7.5 (O.P.)	^48^
Ele-photonic	**3.6**	**None**	**74/56 × 10**^**−6**^ **(E.P.)**	**Our device**

Ele-photonic, E.P. and O.P. stand for electrical-photonic, electrical programing, and optical programing, respectively.

Bold values represent parameters of our devices.

## Discussion

In summary, we have systematically investigated the excitonic/valley-polarized hysteresis of IXs in a WS_2_/WSe_2_ HS. By examining the PL spectra of the WSe_2_ monolayers on hydrophilic and hydrophobic substrates, we verify that the origin of the hysteresis is the chemical-doping of WSe_2_ by O_2_/H_2_O redox couple. Benefiting from the hysteresis effect, IXs can be non-volatilely switched between a spin-singlet state and a spin-triplet state, enabling applications in valley-polarized information processing. Finally, we demonstrate the memory function of the device, which shows a good writing/reading/erasing ability with retention time exceeding 60 min. Our study provides a potential paradigm to achieve nonvolatile valley-addressable memory and thus would greatly advance the development of valleytronic devices.

## Materials and methods

### Sample preparations

Electrodes were fabricated by standard photolithography and thermal evaporation (50 nm/2 nm Au/Cr). The substrates with prefabricated electrodes were ultrasonic cleaned and plasma cleaned for 10 min before the fabrication of the HS. WS_2_ and WSe_2_ monolayer flakes were first mechanically exfoliated onto polymethyl-methacrylate (PMMA) stamps, and then transferred on a SiO_2_ (300 nm)/Si wafer using a dry transfer technique with the aid of an optical microscope and a nano-manipulator. The hydrophobic substrates were prepared via immersing in HDMS vapor for 10 min and then rinsing with acetone for 30 s to form a hydrophobic layer on the substrate^51^. All the samples were not treated by thermal annealing, because this procedure would disable or deteriorate the performance of nonvolatile memory devices. To minimize the generation of interface bubbles, we adopted a tilt-transfer method. The PDMS stamp was tilted for about 2° before transfer, and then pressed down until the upper TMDCs monolayer was approaching the lower one. Afterward, the substrate was heated to 50 °C to advance contact frontier forward further, and finally the heater was turned off when the two TMDCs monolayers were well laminated for about 3 min.

### Optical measurements

The as-fabricated devices were mounted in a continuous flow cryostat with 10^−7^ Torr vacuum. For gate-dependent PL measurement, the sample was excited by a 532 nm laser (23 μW) at 78 K. For the helicity-resolved PL measurement, the sample was excited by a 633 nm laser with a power of 180 μW at 78 K. The time interval between two adjacent spectra is about 1 minute when performing gate-dependent measurements. For the memory operation measurement, the spectra were acquired with *V*_g_ changing cyclically and laser keeping focused on the sample. Each spectrum was measured within 10 s. All the PL spectra were collected by a 50× objective lens (N.A. = 0.7) in a Raman spectrometer (Horiba HR550) with a 600 g/mm grating. A Keithley 2400 sourcemeter was used as the voltage source.

## Supplementary information

Supplementary Information

## Data Availability

The data that support the findings of this study are available from the corresponding author upon request. Supplementary information accompanies the manuscript on the *Light: Science & Applications* website (http://www.nature.com/lsa/).
