# Supplementary material for: Nonvolatile electrical switching of optical and valleytronic properties of interlayer excitons
Source: Light Sci Appl. 2022 Jan 24;11:23. doi: 10.1038/s41377-022-00718-7 (PMC8786835; doi:10.1038/s41377-022-00718-7)
Supplement: Supplementary file 1 — Supplementary Information [file 41377_2022_718_MOESM1_ESM.docx]

Supporting Information for

Nonvolatile Electrical Switching of Optical and Valleytronic Properties of Interlayer Excitons

Tong Ye^1^, Yongzhuo Li^3,4^, Junze Li^1^, Hongzhi Shen^1^, Junwen Ren^1^, Cun-Zheng Ning^3,4,5^, Dehui Li^1,2*^

^1^School of Optical and Electronic Information, Huazhong University of Science and Technology, Wuhan 430074, China

^2^Wuhan National Laboratory for Optoelectronics, Huazhong University of Science and Technology, Wuhan 430074, China

^3^Department of Electronic Engineering, Tsinghua University, 100084 Beijing, China

^4^Frontier Science Center for Quantum Information, 100084 Beijing, China

^5^School of Electrical, Computer, and Energy Engineering, Arizona State University, Tempe, AZ 85287, USA

**Correspondence:** Dehui Li (Email: dehuili@hust.edu.cn, Tel: +86 131 2993 0985)

**Fig. S1. The excitonic hysteresis of the monolayer-WS_2_/bilayer-WSe_2_ (1L/2L) heterostructure in the device. a** The PL spectra of IXs in the monolayer-WS_2_/monolayer-WSe_2_ (1L/1L) and 1L/2L HSs. **b** Power-dependent PL spectra of IXs in the 1L/2L HS. **c** Gate-dependent PL spectra of IXs in 1L/2L HS. **d** PL spectra of IXs in 1L/2L HS at 0V-1, 0V-2 and 0V-3. The 0V-2 spectrum is fitted by double Gaussion peaks.

**Fig. S2. Gate-dependent PL spectra for a stacking-order inversed HS (WSe_2_/WS_2_) on a hydrophilic substrate.** **a** Contour plot for the PL spectra of IXs upon cyclic *V_g_*. **b** PL spectra of the IXs at 0 V with different scanning sequences. **c-e** Schematic diagrams illustrating the Stark effect.

The device exhibits contrary behaviors to that of the WS_2_/WSe_2_ HS shown in Fig. 2a, thus proving the Stark effect. However, this device exhibits weak excitonic hysteresis during the cyclic-*V_g_* sweeping. This might be resulted from the Coulomb screening of electrons that are transferred from WSe_2_ due to the type-II band alignment. These electrons accumulate in WS_2_ and suppress the chemical-doping effect of O_2_/H_2_O molecules.

Fig. S2 c-e illustrate the mechanism of the Stark effect. The interlayer exciton in the WS_2_/WSe_2_ HS can be viewed as an electric dipole pointing upward from WSe_2_ to WS_2_ (Fig. S2 c). At negative gate voltages, the external electrical field points from WS_2_ to WSe_2_ and increases the relative band-offset between WS_2_ and WSe_2_. Consequently, the energy separation between the WS_2_ conduction band and the WSe_2_ valence band is decreased, thus leading to the redshift of the IX emission $\hbar\omega’<\hbar\omega$, as shown in Fig. S2 d and e. Contrarily, the IX peak in the WSe_2_/WS_2_ HS exhibits a blueshift at negative voltages. It is the same case for positive gate voltages.

**Fig. S3. Lorentz (a-c) and Gaussian (d-f) fittings of 0V-1, V-2 and 0V-3 spectra.**

The Lorentz fittings cannot match the experimental data very well (Fig. a-c). The 0V-1 and 0V-3 curves can also be fitted with two Gaussian peaks (Fig. d-f). Nevertheless, unlike 0V-2, the two peaks are located at about 1.36 eV and 1.4 eV respectively. The low-energy peak at 1.36 eV is properly due to the light emission of localized exciton (X_L_) that is trapped by impurity or defect sites in the heterostructures^1,2^. The absence of localized exciton emission at 0V-2 can be attributed to the screening of Coulomb interactions by free carriers^1^.

**Fig. S4. Spin-singlet exciton (IX*^S^*) and spin-triplet exciton (IX*^T^*) and their lifetimes.** **a** Gate-dependent PL spectra of IXs under pulsed laser excitation at 73 K. The spectra are fitted by Gaussion function. The low-energy peak at about 1.3 eV is properly due to localized excitons (IX_L_). **b** Intensity ratio of IX^T^ and IX^S^ (I^T^/I^S^) as a function of *V_g_*. The error bars in **b** represent the uncertainty of the data extraction. **c** Contour plot of time-resolved PL intensity of IXs upon *V_g_*. **d** Time-resolved PL profiles at selected gate voltages. The dashed cyan lines are biexponential fittings. **e** Lifetime of IXs upon cyclic *V_g_*. *τ_1_* and *τ_2_* is the extracted fast and slow timescale, respectively. The sample was excited by a picosecond laser centered at 405 nm wavelength with 20 MHz repetition rate with average power of about 10 μW. A 800 nm (1.55 eV) longpass filter was utilized for blocking high-energy PL emissions in the time-resolved measurements.

**Fig. S5. PL spectra (a) and the corresponding contour plot (b) of the WSe_2_ monolayer under *V_g_* scanning from 0 V to −60 V shown in Fig. 3a.**

The trion peak ($X^{-}$/$X^{+}$) and the neutral exciton ($X_{0}$) evolve non-monotonously as *V_g_* changes (Fig. S5a). The $X^{-}$ peak is gradually suppressed while the $X^{+}$ peak is enhanced. Meanwhile, the $X_{0}$ peak is abnormally strengthened at first and then weakened gradually. Such peculiar features strongly suggest that the WSe_2_ is preliminarily *n*-doped, and gradually become *p*-doped by the efficient gate control. In addition, we can obtain the charge neutrality point of the device from Fig. S5b, which is at about −15 V.

**Fig. S6. The retention time of the chemical-doping state of monolayer WSe_2_ on hydrophilic substrate (WSe_2_/SiO_2_). a** Contour plot for the PL spectra of WSe_2_ upon “write”, “read” and “erase” operations. **b** Time-dependent PL spectra of WSe_2_ after the “write” operation. The measurement was performed at 77 K.

As shown in Fig. S6a, monolayer WSe_2_ is chemically n-doped and the negative trion peak is dominated after “write” operation (−60 V). The chemical-doping state of WSe_2_ can keep within only about 5 minutes, because trapped electrons are gradually released from WSe_2_ after *V*_g_ going back to 0 V (Fig. S6b). In contrast, the negative trion peak is quenched immediately after the “erase” operation (60 V). The time-dependent PL spectra clearly show the different transfer rates of electrons upon “write” and “erase” operations, thus proving the blocking effect of the chemical-potential barrier between WSe_2_ and O_2_/H_2_O. Since the chemical potential of O_2_/H_2_O (–5.3 eV) is slightly higher than the valence band of WSe_2_ (–5.46 eV), a chemical-potential barrier (≈0.16 eV) is formed between WSe_2_ and O_2_/H_2_O (Fig. 3c). It blocks chemically-doped electrons out of WSe_2_ when *V_g_* changes from –60 V to 0 V. On the contrary, the chemically-doped electrons can move out of WSe_2_ very quickly with the aid of *V_g_* changing from 0 V to 60 V. Therefore, the dynamic process (–60 V to 0 V) takes a much longer time than that when gate voltage is changed from 0 V to 60 V.

On the other hand, such short retention time (≈5 min) for monolayer WSe_2_ in comparison to that of HS in the manuscript (>>60 min) proves the important role of the type-II band alignment, which renders free electrons transferring from WSe_2_ to WS_2_ when WSe_2_ is chemically *n*-doped. Therefore, we believe the hysteresis of IXs arises from the synergetic blocking effect of chemical-potential barrier between WSe_2_ and O_2_/H_2_O and the type-II band alignment of the WS_2_/WSe_2_ HS.

**Fig. S7. Gate-dependent PL spectra for a WS_2_/WSe_2_ HS on a hydrophobic substrate. a** Contour plot for the PL spectra of IXs upon cyclic *V_g_*. **b** PL spectra of the IXs at 0 V with different scanning sequences.

The device shows similar behaviors to that of WS_2_/WSe_2_ on hydrophilic substrate shown in Fig. 2a, but with weak hysteresis, thus proving the importance role of O_2_/H_2_O molecules.

**Fig. S8. Gate-dependent PL spectra of a WS_2_/WSe_2_/hBN HS.** The WS_2_/WSe_2_ HS was transferred on a hydrophilic SiO_2_/Si substrate with a region insulated by a thin layer *h*BN as **a** shows. **b,c** Gate-dependent PL spectra of the WS_2_/WSe_2_ HS region. The 0V-2 spectrum is fitted by two Gaussian peaks, which are attributed to the spin-singlet state (purple) and spin-triplet state (pink) of interlayer excitons. **d** Optical micrograph of the device. The inset is the height profile measured along the white dashed line. **e,f** Gate-dependent PL spectra of the WS_2_/WSe_2_/*h*BN HS region. **g,h** Selected PL spectra of another device as shown in the inset.

The hysteresis is observed only in the region where WS_2_/WSe_2_ HS is directly contacted with the hydrophilic substrate, other than the region that is insulated by *h*BN (≈50 nm thick), thus proving the important role of O_2_/H_2_O molecules. The weak intensity deviations of 0V-1, 0V-2 and 0V-3 measured in *h*BN-supported region is not reproducible in different samples and might be due to few electrons that randomly diffuse from the chemical-doped region (WS_2_/WSe_2_/SiO_2_)^3^. Interestingly, the Stark effect is absent in the *h*BN isolated region. This might be due to the staircase at the *h*BN boundary blocking the electrical field from the offset electrode (Fig. S8a), since the electrical field in the HS is inversely proportional to $t_{total}*\frac{\varepsilon_{hBN}}{\varepsilon_{HS}}$, where $t_{total}$is the total thickness of *h*BN and HS.

**Fig. S9. Multi-cycle gate-dependent PL measurements. a** Gate-dependent PL spectra of IXs for two sweeping cycles. **b** Extracted PL intensity of the IX emission peak upon gate voltage. **c,d** Gate-dependent PL spectra of monolayer WSe_2_ in the HS on hydrophilic and hydrophobic substrate, respectively. These samples have been stored in ambient air for over two years, and the measurements were performed with a different experimental setup.

**Fig. S10. Helicity–resolved PL spectra of the WS_2_/WSe_2_ HS. a-d** The helicty-resolved PL spectra upon *V_g_* sweeping from 0 V to −60 V, −60 V ~ 0 V, 0 V ~ 60 V, 60 V ~ 0 V, respectively. The valley-polarization degree can be efficiently modulated by *V_g_*, and shows a strong hysteresis.

**Fig. S11. Electrically controlled memory operation in the HS.** **a** Logarithmic timescale plot of Fig. 5b; **b** Time-dependent gate current and power consumption upon *V_g_*.

The retention time should be much longer than 60 minutes, because both the energy and intensity of IX emission gradually level off as time went by. The current changes exponentially near the edges of writing and reading *V_g_*, suggesting that the dynamic evolution of IXs emission is resulted from charging and discharging processes. The power consumption is the product of *V_g_* and the gate current.





**Fig. S12. Memory performance of the WS_2_/WSe_2_ HS under different temperatures.** **a** Contour plots for the PL spectra under cyclic writing/reading/erasing operations at 150 K, 200 K, 250 K and 300 K, respectively. **b** Circularly polarized PL spectra of IXs under different temperatures. **c** Absolute circular polarization degree of IXs upon temperature. The error bars in **c** represent the uncertainty of the data extraction.

The memory performance of IXs degrades as the temperature increases. Intriguingly, the information encoding ability of the device can host up to about 250 K with good energy difference of the 1 and 0 bits.

**Fig. S13. PL spectra of the 0V-2 and 0V-3 states (a) and the intensity ON/OFF Ratio (b).** The maximum ratio is about 3.6 at 1.38 eV (900 nm), which is larger than peer photonic memories^4-7^.

**References**

1 Vialla, F. *et al.* Tuning of impurity-bound interlayer complexes in a van der Waals heterobilayer. *2D Mater.* **6**, 035032 (2019).

2 Joshi, J. *et al.* Localized Excitons in NbSe2-MoSe2 Heterostructures. *ACS Nano*, **14**, 8528–8538 (2020).

3 Ye, T. *et al.* Room-Temperature Exciton-Based Optoelectronic Switch. *Small* **17**, e2005918 (2021).

4 Farmakidis, N. *et al.* Plasmonic nanogap enhanced phase-change devices with dual electrical-optical functionality. *Sci. Adv.* **5**, eaaw2687 (2019).

5 Ríos, C. *et al.* Integrated all-photonic non-volatile multi-level memory. *Nat. Photonics* **9**, 725–732 (2015).

6 Arjunan, M. S. *et al.* High‐Stability and Low‐Noise Multilevel Switching in In_3_SbTe_2_ Material for Phase Change Photonic Memory Applications. *Phys. Status Solidi RRL* **15**, 2000354 (2020).

7 Zheng, J. *et al.* Nonvolatile Electrically Reconfigurable Integrated Photonic Switch Enabled by a Silicon PIN Diode Heater. *Adv. Mater.* **32**, e2001218 (2020).
